# An Integrated Approach for the Valorization of Sea Bass (*Dicentrarchus labrax*) Side Streams: Evaluation of Contaminants and Development of Antioxidant Protein Extracts by Pressurized Liquid Extraction

**DOI:** 10.3390/foods10030546

**Published:** 2021-03-06

**Authors:** Beatriz de la Fuente, Noelia Pallarés, Francisco J. Barba, Houda Berrada

**Affiliations:** Preventive Medicine and Public Health, Food Science, Toxicology and Forensic Medicine Department, Faculty of Pharmacy, Universitat de València, Avenida. Vicent Andrés Estellés, Burjassot, 46100 València, Spain; beatriz.fuente@uv.es (B.d.l.F.); noelia.pallares@uv.es (N.P.)

**Keywords:** pressurized liquid extraction, sea bass, side streams, protein, SDS-PAGE, antioxidant capacity, mycotoxins, heavy metals

## Abstract

In this study, the presence of As, Hg, Cd, Pb, and mycotoxins in sea bass side streams (muscle, head, viscera, skin, and tailfin) was evaluated as a preliminary step to assess the effect of an innovative extraction technique (Pressurized Liquid Extraction; PLE) to obtain antioxidant protein extracts. Then, a response surface methodology-central composite design was used to evaluate and optimize the PLE extraction factors (pH, temperature, and extraction time) in terms of total protein content and total antioxidant capacity (TEAC and ORAC). Heavy metals were found in all samples while DON mycotoxin only in viscera, both far below the safe limits established by authorities for fish muscle tissue and fish feed, respectively. The selected optimal PLE extraction conditions were pH 7, 20 °C, 5 min for muscle, pH 4, 60 °C, 15 min for heads, pH 7, 50 °C, 15 min for viscera, pH 7, 55 °C, 5 min for skin, and pH 7, 60 °C, 15 min for tailfins. Optimal PLE conditions allowed increasing protein content (1.2–4.5 fold) and antioxidant capacity (1–5 fold) of sea bass side stream extracts compared to controls (conventional extraction). The highest amount of protein was extracted from muscle while the highest protein recovery percentage was found in viscera. Muscle, head, and viscera extracts showed higher antioxidant capacity than skin and tailfin extracts. Moreover, different SDS-PAGE patterns were observed among samples and a greater quantity of protein fragments of lower molecular weight were found in optimal than control extracts.

## 1. Introduction

A large amount of side streams is generated by the food industry during the transformation of raw material into the final commercial product. Particularly, for fish processing industry, fillets are the main product while heads, viscera, skin, fins, trimmings, roes, backbones, etc. are the resulting discards, accounting more than 60% of the total biomass [1]. In addition, the increased demand for fish for human consumption over the last years indicates that the amount of these fish side streams will continue to increase as well as their negative economic and environmental impact [2]. In general, most of the marine rest raw materials (including fish side streams) are considered non-food products and are transformed into animal feed, silage, and fertilizers. However, they contain a considerable amount of high nutritional quality proteins and lipids as well as valuable compounds, which makes them a natural resource to be explored and used, if possible, before being discarded [3,4,5].

European sea bass (*Dicentrarchus labrax*) is one of the most consumed fish in Mediterranean countries, being therefore one of the main species farmed at the European Union (EU). Preventive measures adopted by the EU to reduce the catch of wild sea bass has resulted in an increase in cultured sea bass [6]. In 2017, the production of sea bass from aquaculture accounted for 15% of the total farmed fish worldwide [7]. On the other hand, the growing development of both convenience food items and active packaging has led to a change in the commercialization of fishery products. In this sense, sea bass, which have traditionally been marketed as a complete piece, can be currently found as fillets or eviscerated fish. As a result, there will be an increase in sea bass side streams in the upcoming years. 

The nutritional characterization of several sea bass side streams has been recently evaluated, showing a wide variety of healthy compounds such as unsaturated fatty acids, calcium, phosphorus, manganese, proteins, and amino acids [8]. In addition, Valcarcel et al. [7] developed fish protein hydrolysates from viscera, frames, and trimmings of sea bass and sea bream with in vitro antioxidant and antihypertensive activities, suggesting their use as food additives. Therefore, these studies considered the potential use of sea bass side streams as a raw material to obtain fish compounds to be incorporated into the food industry, thus complying with the circular bioeconomy proposed by the EU [9].

Due to the great interest in the exploitation of fish side streams to obtain high-added-value compounds for the food industry, not only their nutritional composition must be considered, but also their safety. The increase in fish production from the aquaculture sector has also led to a change in the diet of farmed fish. Fish meal and oil have been replaced by different plant-based ingredients, thus contributing to the presence of feedborne mycotoxins in farmed fish [10,11]. Both marine and vegetable ingredients are used for the formulation of European seabass feedstuffs [12], which may cause the transfer of mycotoxins from the plant fraction of the feed to sea bass tissues. Other groups of relevant contaminants within the food chain to be considered in farmed fish are toxic metals such as arsenic, mercury, cadmium, and lead, as these contaminants are usually found in feed, water, and particulate matter in the aquaculture environment [13,14]. Although these contaminants have been traditionally investigated in edible fish tissues, the presence of toxic metals in different side streams of farmed sea bass, sea bream, and meagre have been recently reported [14,15]. Therefore, the safety of any fish side stream should be ensured in order to retain the quality of the product.

It should be noted that the valorization concept is not only based on producing value-added products from unconventional biological resources but it is also related to a sustainable and environmentally friendly approach. This involves finding suitable green technologies to recover valuable compounds from food side streams [16]. In this line, several green extraction techniques such as pulsed electric fields, microwaves, ultrasounds, high pressures, supercritical fluids, pressurised liquid extraction, subcritical water extraction, extrusion assisted extraction, membrane filtration, fermentative extraction, and enzymatic assisted extraction have been considered interesting extraction processes in terms of safety and efficiency to recover valuable compounds from marine discards [17,18,19]. 

Pressurized Liquid Extraction (PLE), also known as accelerated solvent extraction (ASE), has become an alternative technique to extract organic compounds, mainly contaminant substances from environmental, biological, and food materials [20]. Furthermore, the possibility of using non-toxic solvents has promoted the use of PLE as an interesting tool to recover high-added-value compounds from different food matrices and side streams. In addition to this, PLE is appreciated as a fast, easy, and automated extraction process, which makes profit of pressure and temperature to improve the extraction efficiency [21]. According to these advantageous characteristics, different valuable compounds have been extracted from different matrices using PLE. Although most of the studies have been focused on obtaining bioactive compounds from terrestrial vegetable food and related by-products, macro and microalgae also have been used to recover bioactive phytochemicals [22], fatty acids [23], and polysaccharides [24]. Regarding marine discards, the carotenoid astaxanthin and fish oil were successfully extracted by PLE from shrimp head and carapace as well as tuna liver, respectively [19]. However, only red pepper and seaweeds data in relation to protein extraction by PLE were found [4,25]. 

Therefore, this study aims to explore, for the first time, the use of the green PLE technique to obtain antioxidant protein extracts from sea bass processing side streams, in order to give added value to these underutilized residues. For this purpose, muscle left over, heads, viscera, skin, and tailfins were selected after simulating sea bass filleting. The presence of possible farmed fish contaminants for human health (heavy metals and mycotoxins) in these rest raw materials will be also evaluated. Then, the optimal pH–temperature–time combination for PLE-assisted extraction will be determined using the response surface methodology in terms of protein content and antioxidant capacity. The extracts obtained at optimal conditions will be analysed according to their protein fraction (total content and SDS-PAGE pattern for molecular weight distribution) as well as total antioxidant capacity. 

## 2. Materials and Methods

### 2.1. Reagents 

Trolox^®^ (6-hydroxy-2,5,7,8-tetramethylchroman-2-carboxylic acid), ABTS (2,2′-azinobis (3-ethylbenzothiazoline 6-sulfonic acid)), DTT (DL-Dithiothreitol), Trizma^®^ base, fluorescein sodium salt, diatomaceous earth (Hyflo^®^ Super Cel^®^), and formic acid (reagent grade ≥ 95%) were provided by Sigma-Aldrich (Steinheim, Germany). Potassium dihydrogen phosphate, sodium phosphate dibasic, potassium sulfate, sodium chloride, ortho-boric acid, TRIS (ultrapure), AAPH (2,2′-azobis (2-amidinopropane)) (Acros Organics), glycine (proteomics grade), and methanol (HPLC LC-MS grade) were purchased from VWR International Eurolab S.L. (Barcelona, Spain). Sodium hydroxide, glacial acetic acid, and sulfuric acid were supplied by Fisher Scientific (Madrid, Spain). SDS (sodium dodecyl sulfate, purissimum-CODEX) was obtained from Panreac (Barcelona, Spain). Acetonitrile (HPLC grade), acetone, glycerol, and bromophenol blue indicator (ACS reagent) were supplied by Merck (Darmstadt, Germany). Anhydrous magnesium sulfate (99.5% min powder) was provided by Alfa Aesar (Karlsruhe, Germany). Octadecyl C18 sorbent was from Phenomenex (Madrid, Spain) while absolute ethanol was from J.T. Baker (Deventer, The Netherlands). Deionized water (resistivity >18 MΩ cm^−1^) was obtained through a Milli-Q SP^®^ Reagent Water System (Millipore Corporation, Bedford, MA, USA).

### 2.2. Raw Material and Sample Preparation

Whole sea bass fishes (*Dicentrarchus labrax*) were purchased in a local market in Valencia (Spain) during different days of February 2019. According to the commercial label, they were farmed in Burriana (Valencia, Spain). Immediately, they were transported from the market to the University of Valencia under refrigerated conditions.

**Figure 1:**
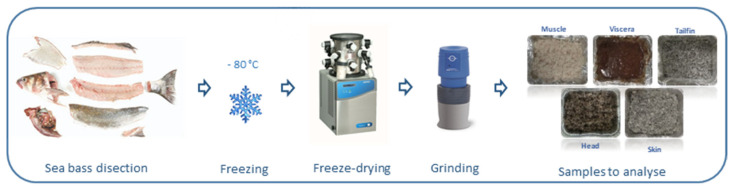
Scheme of sea bass side stream sample preparation.

For sample preparation (Figure 1), each individual sea bass was dissected and different side streams were separated as a simulation of fish processing for human consumption. Then, muscle (white and dark), heads (including gills), viscera, skin, and tailfins were selected and weighed inside aluminum containers before freezing (−80 °C). Frozen samples were lyophilized (LABCONCO, 2.5. FREE ZONE, Kansas City, MO, USA) for 72 h and maintained in a desiccator until constant weight in order to determine their moisture percentage. The moisture values (%) were 65.66 ± 1.55, 51.28 ± 5.08, 21.92 ± 2.28, 34.40 ± 2.37, and 36.44 ± 0.69 for muscle, heads, viscera, skin, and tailfins, respectively), which were in accordance to the values reported by [8]. Next, samples were ground as well as possible in an analytical mill (A11 basic IKA^®^ WERKE, Staufen, Germany). Finally, a pool was made to homogenize each fish side stream before storage at −25 °C until the extraction process and subsequent experiments.

### 2.3. Analysis of Heavy Metals in Sea Bass Side Streams

The presence and content of As, Hg, Cd, and Pb in lyophilized muscle, heads, viscera, skin, and tailfins of sea bass were evaluated. A microwave accelerated reaction system (MARS, CEM, Vertex, Spain) was used for the acid mineralization of samples. According to the side stream, between 0.20 and 0.40 g of sample were placed in a Teflon vessel. Next, 1 mL of H_2_O_2_ (30% *v*/*v*) and 4 mL of HNO_3_ concentrated (64% *v*/*v*) were added to the samples and the digestion was carried out in the microwave system at 800 W and 180 °C for 15 min. After cooling and eliminating the nitrogen vapors, the digested samples were filtered through Whatman No. 1 filter paper and made up to volume with distilled water. Then, an inductively coupled plasma spectrometer mass detector (ICP-MS, Agilent model 7900) was employed to identify and quantify the heavy metals. The operating conditions were as follows: Ar plasma gas flow (15.0 L/min), carrier gas (1.07 L/min), reaction gas (He), nebulizer pump speed (0.10 rps), RF power (1550 W), and RF matching (1.80 V). Internal standard solutions of ^72^Ge, ^103^Rh, and ^193^Ir (ISC Science) at 20 µg/g were used to correct matrix induced signal fluctuations and instrumental drift. 

A standard calibration curve with concentrations ranging from 0–1000 µg/L was used for the quantification of As, Cd, and Pb and while a standard calibration curve between 0 and 100 µg/L was used for Hg. Limits of detection (LOD) were calculated according to the following equation: LOD = 3sB/a where, 3sB is 3 times the standard deviation at zero concentration and a is the slope of the calibration curve. LOD values (µg/L) for each element were As = 0.012, Hg = 0.0015, Cd = 0.0015, and Pb = 0.004. Distilled water was used as a blank and the metal concentrations in the digested blank were subtracted from the sample values. The results were expressed as µg of each element/g of side stream in wet weight. In addition, fish protein powder (Certified Reference Material for Trace Metals DORM-3) was used to confirm the accuracy of the method. It was prepared and analyzed using the same procedure as that followed for the sea bass side streams. The recovery percentages were 98%, 86%, 76%, and 77% for As, Hg, Cd, and Pb, respectively. 

### 2.4. Analysis of Mycotoxins in Sea Bass Rest Raw Material 

Mycotoxins analysis was conducted by High Performance Liquid Chromatography coupled with Electrospray Ionization-Quadrupole-Time of flight-mass spectrometry (LC-ESI-qTOF-MS). An Agilent 1200-LC system (Agilent Technologies, Palo Alto, CA, USA) equipped with a vacuum degasser, binary pump, and autosampler as well as a Gemini^®^ column NX-C18 (3 µM, 150 × 2 mm ID) (Phenomenex) were employed for the chromatographic determinations. The mobile phases consisted of water (A) and acetonitrile (B), both with 0.1% of formic acid. The gradient program was 50% B (0–6 min); 100% B (7–12 min); 50% B (13–20 min). The injection volume was fixed at 5 µL and the flow rate at 0.2 mL/min. Mass spectrometry (MS) analysis was performed using a 6540 Agilent Ultra-High-Definition-Accurate-Mass-q-TOF-MS coupled to the HPLC, equipped with an Agilent Dual Jet Stream electrospray ionization (Dual AJS ESI) interface in positive and negative ionization modes. The analysis conditions were as follows: nitrogen drying gas flow (12.0 L min^−1^); nebulizer pressure (50 psi); drying gas temperature (370 °C); capillary voltage (3500 V); fragmenter voltage (160 V); and scan range (m/z 50–1500). Automatic MS/MS experiments were carried out under the following collision energy values: m/z 100, 30 eV; m/z 500, 35 eV; m/z 1000, 40 eV; and m/z 1500, 45 eV. Mass Hunter Workstation software was used for data acquisition and integration.

The extraction of mycotoxins from the freeze-dried side streams was carried out using the QuEChERS procedure according to Pallarés et al. [26] with some modifications. Depending on the sample, between 2 and 4 g were mixed with 30 mL of acidified water (2% formic acid) and stirred for 30 min in an orbital shaker (IKA KS 260). Next, 10 mL of acetonitrile were added and an additional 30 min shaking was performed. Then, 2 g of NaCl and 8 g of MgSO_4_ were added and vortexed for 30 s before centrifugation at 4000× rpm for 10 min. Afterward, 2 mL of supernatant were transferred into a 15 mL tube containing 0.3 g of MgSO_4_ and 0.1 g of Octadecyl C18 sorbent. The mixture was shaken and centrifuged under the same previous conditions and the supernatant was filtered (13 mm/0.22 μm nylon filter). Finally, 20 μL were injected into the LC-ESI-qTOF-MS system.

### 2.5. Protein Determination

The total nitrogen content was evaluated in sea bass side streams as well as control and PLE extracts using the Kjeldahl method [27]. The protein–nitrogen conversation factor (6.25) used for fish and side streams was applied in order to obtain the total protein content.

### 2.6. Evaluation of Total Antioxidant Capacity

#### 2.6.1. Trolox Equivalent Antioxidant Capacity Assay (TEAC)

The TEAC assay measures the reduction of the radical cation ABTS^+^ by antioxidant compounds. The spectrophotometric method proposed by Barba et al. [28] was used. The ABTS+ radical cation stock solution was generated by chemical reaction with 7 mM ABTS and 140 mM K_2_S_2_O_8_ overnight in darkness at room temperature. Next, it was diluted in ethanol until an absorbance of 0.700 ± 0.020 at 734 nm and 30 °C to obtain the ABTS+ working solution. The optimization of the adequate dilution of the samples to obtain a percentage of absorbance inhibition of approximately 50% was required. Trolox standard solutions were prepared in a range of 0 to 300 μM. The absorbance of 2 mL of ABTS^+^ working solution was considered the initial point of reaction (A_0_). Then, 100 μL of diluted samples or Trolox standards were added immediately. The absorbance after 3 min of reaction was considered the final point (A_f_). All readings were carried out in a thermostatized UV–vis spectrophotometer. The percentages of absorbance inhibition were calculated from the following equation: 1 − (A_f_/A_0_) × 100 and were compared to Trolox standard curve to express the results as μM Trolox Equivalents.

#### 2.6.2. Oxygen Radical Absorbance Capacity Assay (ORAC)

The ORAC assay measures the capacity of the antioxidant compounds to scavenge peroxyl radicals. The fluorimetric method described by Barba et al. [28] was applied. The reaction was carried out at 37 °C in a Multilabel Plate Counter VICTOR3 1420 (PerkinElmer, Turku, Finland) with fluorescence filters for an excitation wavelength of 485 nm and an emission wavelength of 535 nm. Sodium fluorescein and AAPH solutions were used at a final concentration of 0.015 and 120 mg/mL, respectively. Trolox (100 μM) was used as antioxidant standard and samples were properly diluted. All of them were prepared with phosphate buffer (75 mM, pH 7). The final reaction consisted of 50 μL of diluted sample, Trolox standard or phosphate buffer (blank), 50 μL of fluorescein, and 25 μL of AAPH. The fluorescence was recorded every 5 min over 60 min (until the fluorescence in the assay was less than 5% of the initial value). The results were calculated considering the differences of areas under the fluorescence decay curve (AUC) between the blank and the sample over time, and were expressed as μM Trolox Equivalents.

### 2.7. Molecular Weight Distribution of Protein Fragments

The molecular weight distribution of protein in both control (stirring) and optimal (PLE) aqueous extracts from sea bass side streams were investigated by sodium dodecyl sulfate polyacrylamide gel electrophoresis (SDS-PAGE). Fish extracts were mixed with cold acetone (1:4, *v*/*v* ratio) and centrifuged at 11,000× rpm, 4 °C, and 10 min in order to precipitate fish protein. Then, the supernatant was removed and the pellet was dissolved in distilled water assisted by ultrasound (10 min). Next, equal volumes of protein solution and SDS-PAGE sample buffer solution (62.5 mM Tris-HCl (pH 6.8), 2% SDS, 20% glycerol, 0.01% bromophenol blue, and 50 mM dithiothreitol) were mixed and heated at 95 °C for 5 min. After denaturalization, 10 μL of mixture were loaded on the 8–16% Mini-PROTEAN^®^ TGX™ Precast gels (Bio-Rad) and subjected to electrophoresis using a Mini-PROTEAN^®^ tetra cell (Bio-Rad). The running buffer consisted of Trizma^®^ base (25 mM), glycine (192 mM) and SDS (0.1%). The protein fragments separation was performed at a constant voltage of 80 V for 120 min. Finally, electrophoresed gels were stained in 0.125% Coomassie brilliant blue R-250 and destained in 20% methanol and 10% acetic acid until the background was clear. A standard molecular weight of protein bands from 5 to 250 KDa (Precision Plus Protein™, Bio-Rad) was used to estimate the molecular weight of protein bands. The images of the electrophoretic gels were analyzed using the ImageJ^®^ software (Java 1.8.0_112) a public domain digital image processing program developed at the National Institutes of Health (NIH).

### 2.8. Pressurized Liquid Extraction (PLE) Optimization

#### 2.8.1. PLE Extraction Process

The accelerated solvent extractor ASE 200 Dionex (Sunnyvale, CA, USA) equipped with a solvent controller was used for the extraction of water-soluble compounds (protein fraction and antioxidants) from sea bass side streams. Nitrogen (145 psi) was applied to assist the pneumatic system and to purge the cells. Distilled water was used as extracting solvent. The standard operating conditions were as follows: preheating period (1 min), heating period (5 min), flush volume (60%), nitrogen purge (60 s), and extraction pressure (1500 psi). The variable extraction conditions consisted of different ranges of pH (4–10), temperature (20–60 °C), and time (5–15 min). 

All samples were mixed with diatomaceous earth (DE) before the extraction process. Both, the ratio (sample:DE) and the total amount of mixture were previously studied for each side stream. The extractions were performed in 22 mL pressure-resistant stainless steel cells with a glass fiber filter placed in the end part. Each aqueous extract obtained was homogenized, divided into several replicates, and stored at −25 °C for subsequent analyses.

#### 2.8.2. Experimental Design and Optimization of Extraction Conditions

The response surface methodology (RSM) was used to evaluate the effect of selected independent variables (pH, temperature, time) and determine the optimal conditions for the extraction of water-soluble proteins and antioxidant compounds. For this purpose, a central composite design (CCD) was used for the optimization of the extraction conditions. This statistical model provided a total of 16 experiments which were conducted in a randomized order (Table 1). According to the CCD, one of the 16 pH–temperature–time extraction combinations was performed in duplicate in order to check the reproducibility and stability of the results. For the rotatable model, 3 to 5 central points are recommended, while for the centered face model used in this study, 1 to 2 central points are considered sufficient (Statgraphics Centurion XVI.I). In this way, some authors have previously used the same conditions for the CCD model [29,30]. Total protein content and total antioxidant capacity in fish extracts were the responses (dependent variables). The desirability method was used to find a common value for the dependent variables. Surface plots were generated by assigning constant value to one of the three variables studied. In addition, the effect of each independent variable on each of the responses was also studied and the corresponding graphics were created. The analysis was carried out through the Statgraphics Centurion XVI.I. 

After acquiring the theoretical optimal conditions and knowing the impact of single variables on the responses, final PLE extraction conditions were selected and new extracts were obtained. At the same time, conventional extraction (control) was carried out under stirring (30 min) with distilled water at room temperature. Control samples were performed in parallel for all sea bass processing side streams. Then, extra experiments were carried out to investigate the protein fraction (protein content and protein molecular weight distribution) and the total antioxidant capacity in both control and optimal PLE extracts. 

### 2.9. Statistical Analysis

Experimental data were subjected to one-way analysis of variance (ANOVA) to determine the significant differences among samples. Tukey HSD (Honestly Significant Difference) multiple range test, at a significance level of *p* < 0.05 was applied. Statistical analyses were performed with the software Statgraphics Centurion XVI® (Statpoint Technologies, Inc., The Plains, VA, USA).

## 3. Results and Discussion

### 3.1. Determination of Heavy Metals and Mycotoxins in Sea Bass Side Streams

The concentration of heavy metals in sea bass muscle left over, heads, viscera, skin, and tailfins are reported in Table 2. As can be seen in the table, the concentration ranges expressed as µg/g of wet weight (ww) were 0.346–1.867, 0.015–0.106, 0.001–0.028, and 0.027–0.063 for As, Hg, Cd, and Pb, respectively. For all rest raw materials, the most abundant element was As. However, the order of Hg, Cd, and Pb differed according to each side stream. Hg ranked second for muscle and tailfins while Pb ranked second for heads, viscera, and skin. In general, the information available in the literature regarding metal contamination in fish tissues other than those considered edible is scarce. This is because the presence of heavy metals in fish has been only considered as a risk to human health when fish meat was the target sample. In this sense, Renieri et al. [31] determined the levels of Hg, Cd, and Pb in muscle tissue of sea bass and sea bream from different aquaculture sites and fisheries. With respect to sea bass, similar results (µg/g, ww) were reported for Cd (0.001), Pb (0.007–0.138), and Hg (0.022–0.113), with all values far below the safe limits for consumption established by authorities [32]. Regarding different sea bass side streams, Kalantzi et al. [14] investigated the accumulation of metals and trace elements in muscle, liver, gills, bones, and intestines of farmed seabass as well as its correlation with the environmental conditions at the farming sites. It should be noted that the sum of the intestine and liver values would be equivalent to the values of the viscera sample of this study. Likewise, the gills values would correspond to those of the head samples. Thus, the authors found a higher mean arsenic content (µg/g, ww) in muscle (0.867), gills (0.455), and viscera (2.202). On the contrary, the average of Hg concentration was lower for all side streams, being 0.062, 0.001, and 0.040 µg/g (ww) for muscle, gills, and viscera, respectively. Pb was not detected in muscle but the mean values in gills and viscera were 0.035 µg/g (ww) and 0.240 µg/g (ww), respectively. Similarly, there was no presence of Cd in muscle or gills, while the values in viscera were 0.322 µg/g (ww). Although there is no literature data regarding the concentration of metals in sea bass skin and tailfins, a recent study determined several trace elements in different sea bream samples (a specie closely related to sea bass) side streams, including skin [15]. Since the limits for toxic metals in fish side streams are not currently legislated, their assessment could be carried out according to those established for fish muscle. In this sense, the existing regulatory limits for As, Hg, Cd, and Pb are 13.5, 0.5, 0.05, and 0.30 µg/g of wet tissue [15,31], values much higher than those obtained in this study. Therefore, all sea bass side streams analyzed could be considered as safe to be used for the food industry under the circular economy point of view, in terms of As, Hg, Cd, and Pb content.

Regarding mycotoxins, a home-made spectral library containing of 223 mycotoxins and a non-targeted screening approach were applied to investigate the presence of mycotoxins in sea bass tissues. Deoxynivalenol (DON) was identified in viscera sample. LOD and limit of quantification (LOQ) were 0.1 ppb and 0.5 ppb respectively and a standard calibration curve from 0.5 to 1000 ppb was used for DON quantification with regression coefficients higher than 0.9990. Recoveries assays at 5 and 25 ppb were above 85% very similar to those obtained previously [26]. The positive sample showed traces of DON with levels ranging from LOD and LOQ.

DON is a mycotoxin primarily produced by *Fusarium* fungi, occurring mainly in cereal grains used to elaborate feeding for fish. As a result, DON is also known for its high prevalence and incidence in both feed ingredients and feed end-products in Europe [33]. For instance, high concentration of DON in sea bream feeds due to wheat ingredient has been reported [34]. The maximum level of DON allowed by the European Commission in feed material is 12 mg/kg in maize by-products, 8 mg/kg for other cereals, and up to 5 mg/kg for complete and complementary feedstuff [35]. The intake of DON-contaminated feeds can affect not only fish health but also that of the final consumer of the food chain. 

DON is considered to be rapidly metabolized and excreted by fish, thus producing low retention in tissues [33]. However, DON was evenly distributed in muscle, liver, kidney, skin, and brain of Atlantic salmon fed with contaminated feed for 2 months [36]. On the other hand, the estimated mean dietary concentration of DON in different farmed fish species concluded that adverse effects in human health were not expected [37]. Despite this, limit values for mycotoxins in fish tissues should be established by the authorities.

### 3.2. PLE Optimization

The influence of pH, temperature, and time on the development of protein extracts with antioxidant capacity of several sea bass side streams (muscle, heads, viscera, skin, and tailfins) obtained by PLE was studied using a response surface methodology. The experimental values for each independent variable provided from the central composite design and the responses obtained are shown in Table 3. Due to the lack of information on the behavior of the proteins present in fish side streams as well as on their different tissues, the selection criteria for the extraction conditions were based on data from fish muscle proteins and the search for the most sustainable extraction process. It is known that the solubilization of fish muscle proteins depends on the protonation of amino acid residues of the protein side chains, with a mean pH value of 4.0 for aspartyl and glutamyl and 9.9 for lysyl, tyrosyl, and cysteinyl [38]. According to this, acidic (4), neutral (7), and basic (10) pH were selected for all side streams. Since high temperatures may affect thermolabile compounds, mild temperatures (up to 60 °C) were chosen [20,38]. In addition, static extraction cycles of 5 min are required for the ASE equipment used. In order to achieve shorter extraction time, between 1 and 3 extraction cycles were selected.

In addition to the factors of pH, temperature, and extraction time evaluated, other parameters such as pressure and solvent were also studied. The pressure is mainly responsible for maintaining the solvent in a liquid state, and has a limited impact on the extraction efficiency [39,40]. For the extractions carried out with different ASE Dionex models like the one employed in this study, 1500 psi is the constant pressure typically used [21,41,42,43]. Since authorized green solvents are recommended to recover food compounds by PLE, water was used in order to perform an extraction as much sustainable as possible for a future application in the food industry.

#### 3.2.1. Protein Content 

The results of total protein content in sea bass side stream extracts are shown in Table 3. The amount of protein in muscle extracts ranged from 134 mg (pH 10/20 °C/15 min) to 549 mg (pH 7/40 °C/10 min). For head extracts, the results varied from 130 to 342 mg of protein in extract, which corresponded to a combination of factors of pH 10/20 °C/15 min and pH 10/60 °C/15 min, respectively. Regarding viscera extracts, the protein values obtained with different extraction methods were found between 68 and 124 mg for pH 4/20 °C/15 min and pH 7/60 °C/10 min, respectively. As for the skin extracts, the lowest protein content was 73 mg (pH 4/20 °C/5 min) while the highest was 353 mg (pH 7/60°C/10 min). With regard to tailfins, the amount of protein in the extracts ranged from 98 to 298 mg with extraction conditions of pH 7/20 °C /10 min and pH 10/60 °C /15 min. According to these results, the influence of the combination of pH, temperature, and time on protein content in the extracts depended on the fish matrix.

In addition to the extracts, total protein content was also determined in freeze-dried samples in order to know the protein recovery from the sea bass raw materials to the solvent (water) after applying the PLE technique. The percentage of protein in sea bass side streams (dry weight, dw) was 79.18 ± 0.36, 48.08 ± 0.55, 18.24 ± 1.07, 52.14 ± 5.32, and 49.87 ± 0.89 for muscle, heads, viscera, skin, and tailfins, respectively. These results are in close agreement with the values recently reported by Munekata et al. [8] and Valcarcel et al. [7] (except for tailfins that have not been considered in those studies). Regarding protein recovery from fish side streams samples, the percentage was calculated by applying the following equation: (amount of protein in extract/amount of protein in lyophilized sample) × 100. The ranges of protein recovery after applying the different pH–temperature–time combination methods were around 7–28% (muscle), 11–28% (heads), 26–48% (viscera), 7–34% (skin), and 10–30% (tailfins). It should be noted that the best values of protein recovery were observed for viscera extracts, despite being the sea bass side stream with the lowest amount of proteins. In addition to the main factors, there are other important parameters such as the particle size of the sample that can influence the efficiency of the PLE extraction process [39]. In this sense, the skin and tailfin tissues could not be completely grinded and homogenized during sample preparation, so the contact surface for the aqueous extraction was smaller compared to muscle and viscera samples which resulted in lower amount of extracted protein.

##### Effect of Individual pH, Temperature, and Time on Protein Extraction

**Figure 2:**
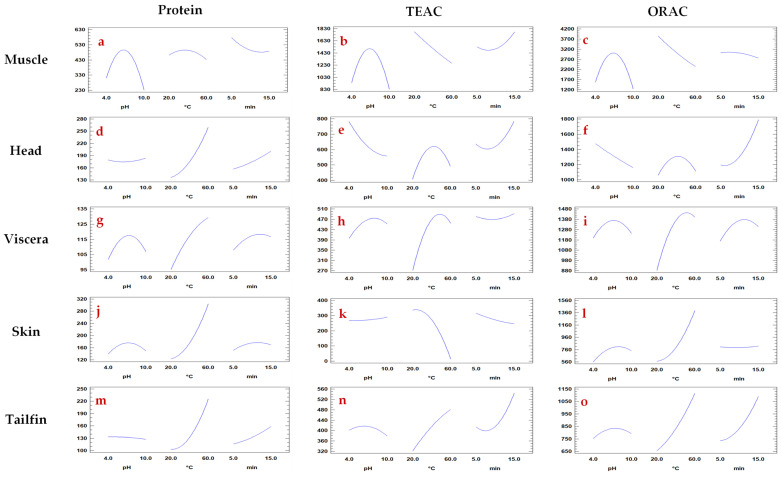
Main effects of each independent variable (pH, temperature, time) of Pressurized Liquid Extraction for each response (total protein content and total antioxidant activity (TEAC and ORAC)) in extracts from seabass muscle (**a**–**c**), head (**d**–**f**), viscera (**g**–**i**), skin (**j**–**l**), and tailfins (**m**–**o**).

The behaviour of each single variable (pH, temperature, and extraction time) on total protein content of each sea bass side stream extract is shown in Figure 2. As can be seen in the Figure 2a–o, the pH value mainly influenced protein extraction from muscle (Figure 2a) and viscera (Figure 2g), while it was not a determining factor for heads, skin and tails (Figure 2d,j,m). Protein extraction is clearly improved by increasing the temperature independently of the sample, except for muscle. On the other hand, a longer extraction time enhanced the amount of proteins obtained from heads, viscera, and tails. However, the time factor was not decisive for skin and muscle samples. In general, temperature and pH were more relevant parameters than extraction time for protein response.

#### 3.2.2. Total Antioxidant Capacity

To evaluate the quality of the obtained extracts, the total antioxidant capacity (TEAC and ORAC assays) was determined (Table 3). A wide variety of antioxidant activity values were observed among the different fish extracts. The values ranged from not detected to 2111 and from 401 to 4572 μM Trolox Eq for TEAC and ORAC assays, respectively. For muscle samples, the antiradical activity ranged from 620 to 2111 μM Trolox Eq (TEAC) and from 511 to 4572 μM Trolox Eq (ORAC). Regardless of the extraction time, the lowest values were found at 60 °C for all pH values studied. The antioxidant capacity in head extracts varied from 335 to 986 μM Trolox Eq (TEAC) and from 571 to 1949 μM Trolox Eq (ORAC). Regarding the remaining sea bass side streams (viscera, skin, and tails), the antioxidant capacity values were approximately below 600 and 1500 μM Trolox Eq for TEAC and ORAC tests, respectively. The highest values of antioxidant capacity were found in muscle sample, which seem to be related to the solvent neutral pH. It should be noted that for head, viscera, and tailfin extracts, both mechanisms of antioxidant action showed a similar behavior between the different combinations of pH, temperature, and extraction time used. However, with regard to the skin sample, the extraction method applied at 60 °C negatively affected the antioxidant capacity of the extract components regardless of pH and extraction time. There is a great variety of phytochemicals with in vitro antioxidant capacity extracted by PLE from plant products and by-products [21,44,45]. However, as far as we know, there are no examples in the literature on the application of PLE to recover food compounds from food of animal origin and related side streams.

##### Effect of Individual pH, Temperature, and Time on Antioxidant Capacity

The behavior of each single variable (pH, temperature, and extraction time) on total antioxidant capacity (TEAC and ORAC) of sea bass side stream extracts is shown in Figure 2. Although these assays measure the antioxidant capacity of compounds through different mechanisms of action, no differences were observed in the pH influence on the antioxidant activity for each side stream and method used. Moreover, the effect of temperature on the antioxidant capacity (TEAC and ORAC) was similar for each muscle (Figure 2b,c), head (Figure 2e,f), viscera (Figure 2h,i), and tailfin (Figure 2n,o) extracts. Regarding skin, the increase in temperature caused both the decrease of TEAC (Figure 2k) values and the increase of ORAC (Figure 2l) values of the extracts. Differences in the response of antioxidant capacity based on temperature factor have also been observed in extracts of chestnut shell and bean [46,47]. The extraction time had a similar impact on the antioxidant capacity (TEAC and ORAC) of fish extracts for head (Figure 2e,f), skin (Figure 2k,f), and tailfin (Figure 2n,o) samples. However, different behavior was observed for muscle (Figure 2b,c) and viscera (Figure 2h,i). For instance, increased extraction time augmented TEAC and slightly decreased the ORAC values. Moreover, in general, it was found that the in vitro antioxidant properties of the fish extracts were less influenced by pH factor.

#### 3.2.3. Effect of the pH–Temperature–Time Combination on Common Response

**Figure 3:**
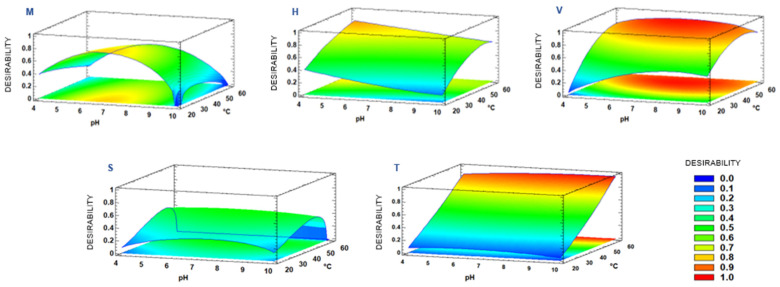
Estimated response surface by plotting desirability versus pH (4–10), temperature (20–60 °C), and 15 min of extraction time for each sea bass side stream (M: Muscle, H: Head, V: Viscera, S: Skin, T: Tailfin). Desirability is based on the joint response of the different responses analyzed (total protein content and total antioxidant capacity by TEAC and ORAC assays).

Graphical analysis in terms of response surfaces was performed in order to visually interpret the effect of the combination of the different PLE extraction variables (pH, temperature, and time) on the different analyzed responses (protein content and antioxidant capacity by TEAC and ORAC). It should be noted that the optimization was carried out on the basis of both the protein content values and the antioxidant capacity (TEAC and ORAC) values. For this purpose, the desirability method was used as a common value to the three responses and then the response surface method provided the combination of the experimental factors that simultaneously optimizes several responses (by maximizing the desirability). According to this, Figure 3 shows the response surface plots of the effect of the combination of PLE extraction variables on protein content and antioxidant capacity in the fish extracts obtained. 

The predicted optimal extraction conditions (pH/°C/min) were 6.8/20 °C/5.0 min for muscle left over, 4.0/60 °C/15.0 min for heads, 7.3/49 °C/12.7 min for viscera, 7.7/53 °C/5.0 min for skin, and 9.4/59 °C/15.0 min for tailfins. However, the ASE equipment does not allow entering a temperature value other than an exact number. In the same way, the static extraction mode used by PLE is based on 5 min cycles, thus reducing the selection of extraction time. Taking into account these technical limitations, the optimal PLE conditions selected were (pH/°C/min): 7/20/5 for muscle, 4/60/15 for heads, 7/50/15 for viscera, 7/55/5 for skin, and 7/60/15 for tailfins. 

After verifying that the pH factor did not significantly influence protein content and antioxidant capacity in tailfin extracts (Figure 2m–o), neutral pH instead of basic pH was selected for this sample. It should be noted that 59 and 60 °C were the optimum temperatures for tailfin and head, respectively. Since the antioxidant capacity (TEAC and ORAC) decreased at 60 °C (Figure 2e,f), a higher temperature could affect even negatively the antioxidant compounds of head extracts. In addition to this, extraction time of 25 min was explored following the trending of 15 min as optimal extraction for some head, viscera, and tailfin samples. However, no higher values were obtained either for protein or antioxidant capacity compared to 15 min extractions (data not shown). Noteworthy that the optimum extraction time for two of the five side streams studied was 5 min, confirming PLE as a fast technique.

### 3.3. Evaluation of RSM Mode

The RSM model provided a regression equation fitted to the experimental data and specific for each response and side stream (a total of 15 equations). The substitution of the pH, temperature, and time values into the equations resulted in a theoretical value for each response according to the CCD model. In general, the predicted and experimental values were similar for all pH–temperature–time combinations and samples. In addition, the reproducibility of the results was verified from the values obtained in the central points of the CCD model. Good coefficients of variation were obtained for protein content, TEAC and ORAC for all side streams, except for the antioxidant capacity values of the tailfin extracts. Experimental and predicted data for the response variables obtained from the CCD for sea bass muscle, head, viscera, skin, and tailfin extracts are reported in Supplementary Materials (Tables S1–S5). All the regression equations required for the calculations (Equations (S1)–(S15)) as well as the coefficient of variation of the central point from the CCD model (Table S6) are also included. Since maximal protein content, TEAC, and ORAC values were achieved individually at different extraction parameter combinations, the RSM method was applied to optimize the PLE conditions for these three responses together. For this purpose, the desirability (d) function approach was used. It is a multiplicative model of individual desirability that provides a desirability scale ranging from 0 to 1 [48]. The ideal optimum value is d = 1 and an acceptable value is 0.6 < d < 0.8. According to the RSM method, desirability values of 0.8 were obtained for muscle and head, 0.9 for viscera and tails, and 0.6 for skin.

### 3.4. Optimal PLE Extracts and Comparison to Control Extracts

Both the extracts obtained by PLE after applying the selected optimal extraction conditions and the extracts obtained by conventional stirring (control) were characterized based on total protein content and molecular weight distribution of protein fragments as well as the total antioxidant capacity.

#### 3.4.1. Total Protein Content

**Figure 4:**
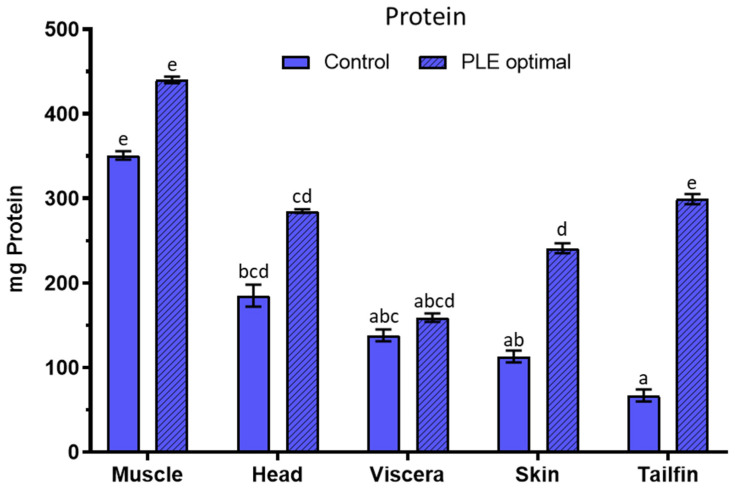
Total protein content in control extracts and optimal PLE extracts from sea bass muscle, head, viscera, skin, and tailfin side streams. PLE: Pressurized Liquid Extraction. Results are expressed as mean ± standard deviation (*n* = 2). Different lowercase letters in the bars indicate statistically significant differences (*p* < 0.05) among samples.

The results of the amount of protein extracted in optimal and control extracts of all sea bass side streams are shown in Figure 4. The milligrams of protein in the optimal extracts of sea bass muscle, head, viscera, skin, and tailfins were 440 ± 4, 285 ± 2, 159 ± 5, 241 ± 6, and 299 ± 6, respectively, while they were 351 ± 5, 185 ± 13, 138 ± 7, 113 ± 7, and 67 ± 7 in their corresponding control extracts. Therefore, PLE-assisted extraction improved by 1.2 to 4.5 times (depending on the sample) the protein extraction of sea bass side streams in one sixth and half the time compared to controls (5 and 15 vs. 30 min). Since neutral pH water was used for most of the fish side streams in both PLE extraction and traditional stirring, it could be concluded that pressure and temperature played an important role in the extraction process. The protein recovery percentages were calculated around 22, 18, 61, 18, and 30% for optimal extracts of sea bass muscle, head, viscera, skin, and tailfins respectively whilst 18, 15, 52, 11, and 14% for controls. As far as we know, no studies about protein extraction from fish side streams using PLE technique have been reported. Marine matrices (red, green, and brown seaweeds) were used to compare PLE with different protein extraction methods [4]. The application of a 50% methanol–water mixture at 37 °C resulted in a protein recovery of less than 5%, a lower percentage than in this study. In addition, the optimal protein extraction from red pepper seed meal was 12% after applying PLE technique [25].

#### 3.4.2. Protein Molecular Weight Distribution

**Figure 5:**
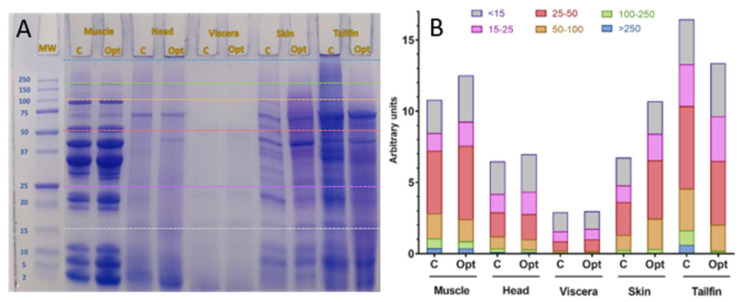
Molecular weight distribution of protein fraction of sea bass side stream extracts. SDS-PAGE electrophoresis patterns (**A**) and grouping of band areas by molecular weight ranges (**B**). MW: Molecular weight standard. C: Control extract. Opt: Optimal extract.

The electrophoretic pattern of sea bass muscle, heads, viscera, skin, and tailfins extracts obtained both by conventional stirring and PLE is shown in Figure 5A. As expected, different protein molecular weight (MW) distribution profiles were observed among the samples due to the different components of each sea bass side stream. In order to achieve a better approximation of the kDa values corresponding to each band compared to the MW standard, the image of the electrophoresis gel was evaluated by the ImageJ Program. In addition, the superposition of the electrophoretic images (standard-sample) allowed grouping in MW intervals the areas of the different bands of each sample (Figure 5B). 

Although visually no great differences were observed in the electrophoretic profiles of controls compared to the optimal ones, the analysis of the images revealed that optimal PLE extracts contained a greater amount of protein fragments of lower MW than control extracts. Therefore, the PLE extraction conditions selected in this study could influence the characteristics of the protein fragments obtained in sea bass side stream extracts.

The muscle proteins (opt) exhibited bands of MW from 9 to 126 kDa while those of the muscle control extract were from 9 to 146 kDa. The clearest bands were found between 25 and 100 kDa in both extracts, in agreement with the electrophoretic profiles of sarcoplasmic proteins from striped catfish and carp meat [49].

Few protein bands ranging from 10 to 85 kDa were observed for both control and optimal head extracts which was in a similar range to that obtained in parrotfish head protein hydrolysates [50]. Poorly defined bands were obtained in viscera samples. The range of MW between 7 and 60 kDa was the same for both extracts. However, a 110 kDa band in the control extract was not found at the optimum. This SDS-PAGE pattern of seabass viscera proteins (8, 18, 27, 49, 60 kDa) was similar to that of undigested cod viscera proteins reported by Aspmo et al. [51]. The presence of protein fragments below 5 kDa could also be observed in both profiles which could be possibly reported as viscera enzymes. Several bands (8–100 kDa) were found both in the controls and in the optimal sea bass skin extracts. The bands corresponding to 85, 46, and 38 kDa of the control extract were not found in the optimal PLE extract, which may be due to the difference in the extraction conditions. The SDS-PAGE technique is usually used to examine collagen or gelatin proteins obtained from fish skin. In this sense, the electrophoretic profiles of hoki and rainbow trout skin gelatin did not correspond to the profiles of the sea bass skin extracts [52,53]. MW of protein fragments of optimal sea bass tailfin extracts ranged between 8 and 80 kDa while those of the controls ranged between 8 and 141 kDa. The two bands with the highest molecular weight (101 and 141 kDa) as well as the 39 kDa band of the control extract did not appear in the electrophoretic profile of the optimal extract, showing again that the extraction parameters could influence the protein fraction.

#### 3.4.3. Total Antioxidant Capacity

**Figure 6:**
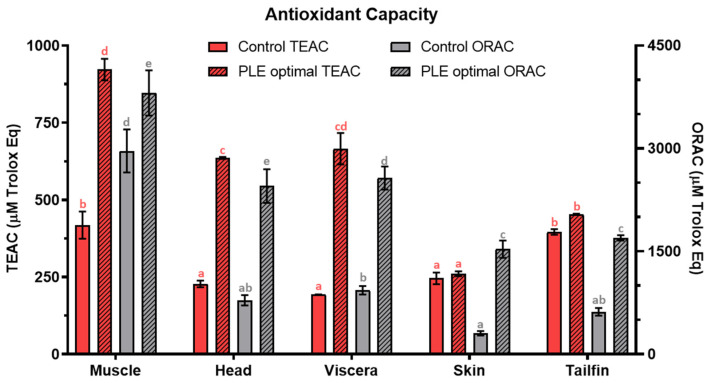
Total antioxidant capacity (TEAC and ORAC) in control extracts and optimal PLE extracts from sea bass muscle, head, viscera, skin, and tailfin side streams. TEAC: Trolox equivalent antioxidant capacity. ORAC: Oxygen radical absorbance capacity. PLE: Pressurized Liquid Extraction. Results are expressed as mean ± standard deviation (*n* = 3 for TEAC and *n* = 6 for ORAC). Different lowercase letters in the bars indicate statistically significant differences (*p* < 0.05) among samples.

The results of total antioxidant capacity in optimal PLE and control extracts of all sea bass side stream extracts are shown in Figure 6. Total antioxidant capacity measured by both TEAC and ORAC assays was also higher in optimal samples than control extracts for all fish side streams. 

TEAC values were 922 ± 35, 636 ± 37, 666 ± 51, 261 ± 8, and 453 ± 2 µM Trolox Eq for optimal muscle, head, viscera, skin, and tailfins extracts, respectively while values of 418 ± 44, 228 ± 11, 193 ± 1, 246 ± 19, and 396 ± 9 µM Trolox Eq were found for the corresponding controls. Similarly, optimal ORAC values (µM Trolox Eq) were 3808 ± 33 (muscle), 2452 ± 25 (heads), 2569 ± 17 (viscera), 1531 ± 13 (skin), and 1696 ± 39 (tailfins) whereas control ORAC values were 2963 ± 31, 787 ± 76, 934 ± 32, 306 ± 32, and 619 ± 56, respectively. According to these results, the application of PLE improved the antioxidant capacity of all sea bass side stream extracts. Comparing data of control extracts with the optimal ones, the antioxidant capacity increased by 120, 179, 245, 6, and 14% (TEAC) and by 29, 211, 175, 400, and 174% (ORAC) for muscle, heads, viscera, skin, and tailfins, respectively.

Phytochemical compounds such as polyphenols, carotenoids, anthocyanins, etc. from plant foods and their residues are usually considered responsible for their antioxidant capacity. In the case of foods from animal origins and related side streams, the antioxidant properties have been attributed to amino acids, peptides, and proteins [54]. For instance, protein hydrolysates and peptides from several fish processing side streams have shown antioxidant activities and they have been considered as potential substitutes of synthetic antioxidants for the food industry [1,55,56]. Both protein chain size and composition of amino acids are considered key in the antioxidant activity exhibited by protein fragments. In this sense, hydrophobic amino acids and proline, methionine, tyrosine, histidine, lysine, and cysteine may improve the efficiency of antioxidant peptides [1]. Recently, the amino acid profile of several side streams from farmed sea bass have been reported [8]. According to the results, lysine, proline, and tyrosine were determined in muscle, head, gills, guts, liver, and skin while methionine was not detected in guts and gills. Histidine was also not detected in guts.

## 4. Conclusions

Pressurized Liquid Extraction (PLE) is presented here as an interesting tool to obtain protein extracts with antioxidant capacity from sea bass processing side streams. Since the optimal pH value for four of the five samples was close to 7, the PLE-assisted recovery of high-added-value compounds from sea bass side streams could be performed in a sustainable way. Optimal pH–temperature–time combinations allowed to obtain higher total protein content and total antioxidant capacity in PLE extracts. Muscle, head, and viscera optimal extracts showed better total antioxidant capacity (TEAC and ORAC) than skin and tailfin extracts. The highest amount of protein was recovered from sea bass muscle left over while the highest protein recovery percentage (61%) was observed in viscera. Furthermore, the SDS-PAGE pattern of each extract revealed a specific protein molecular weight distribution for each sea bass side stream. Recovering proteins from natural underexploited resources in a sustainable way is one of the H2020 challenges. Finally, this is the first step towards a possible sustainable application of PLE technique to obtain antioxidant protein extracts from fish side streams. Further research is encouraged in this direction to convert fish side stream materials into nutritional and bioactive ingredients for food, feed, and other high-value market.

## Figures and Tables

**Table 1:** Central composite experiment.

Run	pH (X_1_)	Tª (°C) (X_2_)	Time (min) (X_3_)
1	4	60	5
2 ^a^	7	40	10
3	4	20	15
4	10	40	10
5	7	60	10
6	10	60	5
7	10	60	15
8 ^a^	7	40	10
9	4	20	5
10	7	20	10
11	10	20	5
12	7	40	5
13	7	40	15
14	4	60	15
15	4	40	10
16	10	20	15

^a^ central point.

**Table 2:** Concentration (µg/g) of As, Hg, Pb, and Cd in sea bass side streams.

Sea Bass Side Streams	Heavy Metals (µg/g of Wet Weight)			
	As	Hg	Cd	Pb
Muscle	0.687 ± 0.004	0.106 ± 0.001	0.001 ± 0.00001	0.027 ± 0.0002
Head	0.346 ± 0.003	0.034 ± 0.0004	0.003 ± 0.0001	0.063 ± 0.010
Viscera	1.86 7 ± 0.0005	0.014 ± 0.0003	0.028 ± 0.0003	0.046 ± 0.0004
Skin	0.387 ± 0.004	0.026 ± 0.0006	0.004 ± 0.0002	0.040 ± 0.0004
Tailfin	0.388 ± 0.006	0.042 ± 0.0006	0.001 ± 0.0002	0.033 ± 0.0004
[Legislation *]	<13.5	<0.50	<0.05	<0.30

* Values referred to fish muscle tissue ([15,31,32]).

**Table 3:** Results of total protein content and total antioxidant capacity (TEAC and ORAC) in sea bass side stream extracts obtained by Pressurized Liquid Extraction according to the response surface methodology-central composite design.

RSM				Muscle			Head			Viscera			Skin			Tailfin		
Run	pH	Tª	Time	Protein	TEAC	ORAC	Protein	TEAC	ORAC	Protein	TEAC	ORAC	Protein	TEAC	ORAC	Protein	TEAC	ORAC
		(°C)	(min)	mg	µM Trolox Eq		mg	µM Trolox Eq		mg	µM Trolox Eq		mg	µM Trolox Eq		mg	µM Trolox Eq	
1	4	60	5	242	1140	918	213	531	952	101	432	1195	214	129	1124	181	392	816
2	7	40	10	549	1356	3058	161	622	1342	117	507	1440	153	403	686	130	422	918
3	4	20	15	226	1273	1465	162	594	1129	68	156	401	79	372	362	105	400	829
4	10	40	10	207	1003	941	172	335	1005	103	450	1260	149	346	691	116	357	749
5	7	60	10	386	1166	1665	245	365	1210	125	464	1333	353	nd	1517	231	512	1177
6	10	60	5	301	621	705	253	605	839	108	392	981	252	nd	1228	168	445	914
7	10	60	15	178	688	512	342	537	1540	116	430	1050	249	nd	1407	298	608	1501
8	7	40	10	540	1222	2837	189	650	1379	115	474	1241	166	381	749	134	345	723
9	4	20	5	454	1166	2428	149	503	1781	69	220	436	74	298	264	121	357	597
10	7	20	10	456	2079	4572	151	514	911	101	240	978	90	323	525	98	323	605
11	10	20	5	301	955	2241	132	531	571	78	267	520	92	413	717	102	315	662
12	7	40	5	504	1407	2779	156	539	983	111	479	1212	153	376	881	130	399	660
13	7	40	15	510	2111	3060	201	861	1949	115	472	1290	184	159	817	145	592	1179
14	4	60	15	312	859	1016	289	986	1794	124	434	1320	267	nd	1204	264	601	1253
15	4	40	10	285	981	1933	192	986	1576	107	376	1209	155	185	685	146	454	806
16	10	20	15	135	1593	1647	130	469	1700	87	357	968	92	336	487	103	363	663

RSM: response surface methodology; TEAC: Trolox equivalent antioxidant capacity; ORAC: oxygen radical absorbance capacity; nd: not detected.

## Data Availability

Not applicable.

## Supplementary Materials

The following are available online at https://www.mdpi.com/2304-8158/10/3/546/s1, Tables S1–S5: Experimental and predicted data for the response variables obtained from the central composite design for sea bass muscle (S1), head (S2), viscera (S3), skin (S4), and tailfin (S5) extracts; Table S6: Reproducibility of the results according to the coefficient of variation from the values obtained in the central points of the central composite design model for sea bass side stream extracts. Equations (S1)–(S15): Regression equations provided by response surface methodology and Statgraphics Centurion XVI.I for muscle (S1–S3), head (S4–S6), viscera (S7–S9), skin (S10–S12), and tailfin (S12–S15) extracts.
