# Supplementary material for: An Integrated Approach for the Valorization of Sea Bass (Dicentrarchus labrax) Side Streams: Evaluation of Contaminants and Development of Antioxidant Protein Extracts by Pressurized Liquid Extraction
Source: Foods. 2021 Mar 6;10(3):546. doi: 10.3390/foods10030546 (PMC8000804; doi:10.3390/foods10030546)
Supplement: Supplementary file 1 [file foods-10-00546-s001.pdf]

Table S1. Experimental and predicted data for the response variables obtained from the central composite design for sea bass muscle.

RSM				MUSCLE					
Run	pH	Tª (°C)	Time (min)	Protein (mg)		TEAC (µM Trolox Eq)		ORAC (µM Trolox Eq)	
				experimental	predicted	experimental	predicted	experimental	Predicted
1	7	20	10	456	461	2079	1777	4572	3843
2	7	40	5	504	576	1407	1531	2779	3027
3	7	40	10	549	494	1356	1498	3058	3006
4	7	40	10	540	494	1222	1498	2837	3006
5	7	40	15	510	488	2111	1778	3060	2753
6	7	60	10	386	431	1166	1259	1665	2335
7	4	20	5	454	408	1166	1145	2428	2540
8	4	20	15	226	268	1273	1412	1465	1881
9	4	40	10	285	311	981	943	1933	1579
10	4	60	5	242	251	1140	1066	918	845
11	4	60	15	312	280	859	853	1016	917
12	10	20	5	301	320	955	1013	2241	2355
13	10	20	15	135	113	1593	1719	1647	1735
14	10	40	10	207	231	1003	832	941	1236
15	10	60	5	301	246	621	534	705	304
16	10	60	15	178	210	688	762	512	415

Regression equations provided by Response Surface Methodology and Statgraphics Centurion XVI.I for sea bass muscle.

Equation S1: Protein = -351,446 + 329,988*pH + 2,05575*Tª (ºC) - 49,0105*Time (min) - 24,7399*pH^2 + 0,351208*pH*Tª (ºC) - 1,09267*pH*Time (min) - 0,119173*Tª (ºC)^2 + 0,426025*Tª (ºC)*Time (min) + 1,53923*Time (min)^2

Equation S2: TEAC = -1154,72 + 924,751*pH + 6,70513*Tª (ºC) - 103,388*Time (min) - 67,8735*pH^2 - 1,66194*pH*Tª (ºC) + 7,33508*pH*Time (min) + 0,049372*Tª (ºC)^2 - 1,19826*Tª (ºC)*Time (min) + 6,23355*Time (min)^2

Equation S3: ORAC = -3289,88 + 2482,73*pH - 62,1289*Tª (ºC) - 12,0103*Time (min) - 177,646*pH^2 - 1,48406*pH*Tª (ºC) + 0,65075*pH*Time (min) + 0,207019*Tª (ºC)^2 + 1,82696*Tª (ºC)*Time (min) - 4,6517*Time (min)^2

Table S2. Experimental and predicted data for the response variables obtained from the central composite design for sea bass head.

RSM				HEAD					
**Run**	**pH**	**Tª (°C)**	**Time (min)**	Protein (mg)		TEAC (µM Trolox Eq)		ORAC (µM Trolox Eq)	
				experimental	predicted	experimental	predicted	experimental	predicted
**1**	**7**	**20**	**10**	151	136	514	407	911	1063
**2**	**7**	**40**	**5**	156	157	539	635	983	1194
**3**	**7**	**40**	**10**	161	175	622	618	1342	1307
**4**	**7**	**40**	**10**	189	175	650	618	1379	1307
**5**	**7**	**40**	**15**	201	201	861	783	1949	1791
**6**	**7**	**60**	**10**	245	260	365	490	1210	1112
**7**	**4**	**20**	**5**	149	160	503	515	1781	1489
**8**	**4**	**20**	**15**	162	165	594	742	1129	1409
**9**	**4**	**40**	**10**	192	179	986	782	1576	1474
**10**	**4**	**60**	**5**	213	209	531	578	952	1203
**11**	**4**	**60**	**15**	289	292	986	984	1794	1657
**12**	**10**	**20**	**5**	132	129	531	529	571	695
**13**	**10**	**20**	**15**	130	134	469	418	1700	1436
**14**	**10**	**40**	**10**	172	184	335	558	1005	1159
**15**	**10**	**60**	**5**	253	249	605	453	839	545
**16**	**10**	**60**	**15**	342	332	537	521	1540	1819

Regression equations provided by Response Surface Methodology and Statgraphics Centurion XVI.I for sea bass head.

Equation S4: Protein = 307,374 - 21,7314*pH - 5,53485*Tª (ºC) - 6,51543*Time (min) + 0,767816*pH^2 + 0,296229*pH*Tª (ºC) - 0,00725*pH*Time (min) + 0,0577384*Tª (ºC)^2 + 0,193313*Tª (ºC)*Time (min) + 0,162614*Time (min)^2

Equation S5: TEAC = 244,619 - 38,9942*pH + 35,4742*Tª (ºC) - 36,8202*Time (min) + 5,79935*pH^2 - 0,580958*pH*Tª (ºC) - 5,63667*pH*Time (min) - 0,422627*Tª (ºC)^2 + 0,447375*Tª (ºC)*Time (min) + 3,65617*Time (min)^2

Equation S6: ORAC = 2588,45 - 226,216*pH + 27,9453*Tª (ºC) - 237,431*Time (min) + 1,02586*pH^2 + 0,5635*pH*Tª (ºC) + 13,6735*pH*Time (min) - 0,550043*Tª (ºC)^2 + 1,33307*Tª (ºC)*Time (min) + 7,40531*Time (min)^2

Table S3. Experimental and predicted data for the response variables obtained from the central composite design for sea bass viscera.

RSM				VISCERA					
Run	pH	Tª (°C)	Time (min)	Protein (mg)		TEAC (µM Trolox Eq)		ORAC (µM Trolox Eq)	
				experimental	predicted	experimental	predicted	experimental	predicted
1	7	20	10	101	95	240	272	978	884
2	7	40	5	111	108	479	480	1212	1169
3	7	40	10	117	117	507	470	1440	1368
4	7	40	10	115	117	474	470	1241	1368
5	7	40	15	115	117	472	492	1290	1306
6	7	60	10	125	129	464	454	1333	1399
7	4	20	5	69	69	220	198	436	399
8	4	20	15	68	73	156	158	401	484
9	4	40	10	107	102	376	396	1209	1199
10	4	60	5	101	105	432	450	1195	1252
11	4	60	15	124	120	434	417	1320	1228
12	10	20	5	78	82	267	279	520	619
13	10	20	15	87	84	357	334	968	918
14	10	40	10	103	107	450	452	1260	1242
15	10	60	5	108	103	392	384	981	905
16	10	60	15	116	117	430	447	1050	1095

Regression equations provided by Response Surface Methodology and Statgraphics Centurion XVI.I for sea bass viscera.

Equation S7: Protein = -51,906 + 23,9487*pH + 2,02711*Tª (ºC) + 4,13325*Time (min) - 1,45167*pH^2 - 0,0608125*pH*Tª (ºC) - 0,03725*pH*Time (min) - 0,0128375*Tª (ºC)^2 + 0,0277625*Tª (ºC)*Time (min) - 0,2054*Time (min)^2

Equation S8: TEAC = -453,438 + 89,2597*pH + 30,0081*Tª (ºC) - 23,7856*Time (min) - 5,10586*pH^2 - 0,609729*pH*Tª (ºC) + 1,58758*pH*Time (min) - 0,266932*Tª (ºC)^2 + 0,0176625*Tª (ºC)*Time (min) + 0,65629*Time (min)^2

Equation S9: ORAC = -2093,27 + 296,113*pH + 77,5411*Tª (ºC) + 104,62*Time (min) - 16,4249*pH^2 - 2,36308*pH*Tª (ºC) + 3,563*pH*Time (min) - 0,567536*Tª (ºC)^2 - 0,272625*Tª (ºC)*Time (min) - 5,24757*Time (min)^2

Table S4. Experimental and predicted data for the response variables obtained from the central composite design for sea bass skin.

RSM				SKIN					
Run	pH	Tª (°C)	Time (min)	Protein (mg)		TEAC (µM Trolox Eq)		ORAC (µM Trolox Eq)	
				experimental	predicted	experimental	predicted	experimental	predicted
1	7	20	10	90	123	323	336	525	571
2	7	40	5	153	152	376	315	881	805
3	7	40	10	153	176	403	271	686	793
4	7	40	10	166	176	381	271	749	793
5	7	40	15	184	169	159	245	817	818
6	7	60	10	353	304	nd	13	1517	1396
7	4	20	5	74	59	298	331	264	337
8	4	20	15	79	81	372	298	362	309
9	4	40	10	155	140	185	267	685	561
10	4	60	5	214	232	129	92	1124	1132
11	4	60	15	267	277	nd	-4	1204	1299
12	10	20	5	92	87	413	411	717	640
13	10	20	15	92	77	336	367	487	498
14	10	40	10	149	149	346	290	691	739
15	10	60	5	252	255	nd	68	1228	1300
16	10	60	15	249	267	nd	-39	1407	1353

Regression equations provided by Response Surface Methodology and Statgraphics Centurion XVI.I for sea bass skin.

Equation S10: Protein = -135,323 + 56,624*pH - 3,47822*Tª (ºC) + 15,4937*Time (min) - 3,49216*pH^2 - 0,0224375*pH*Tª (ºC) - 0,529917*pH*Time (min) + 0,0951888*Tª (ºC)^2 + 0,0558375*Tª (ºC)*Time (min) - 0,613979*Time (min)^2

Equation S11: TEAC = 126,966 + 11,7984*pH + 15,945*Tª (ºC) - 6,55872*Time (min) + 0,797184*pH^2 - 0,435125*pH*Tª (ºC) - 0,183*pH*Time (min) - 0,242376*Tª (ºC)^2 - 0,1574*Tª (ºC)*Time (min) + 0,357386*Time (min)^2

Equation S12: ORAC = -294,209 + 294,055*pH - 18,3493*Tª (ºC) - 19,5601*Time (min) - 15,9097*pH^2 - 0,563375*pH*Tª (ºC) - 1,905*pH*Time (min) + 0,475495*Tª (ºC)^2 + 0,48815*Tª (ºC)*Time (min) + 0,731917*Time (min)^2

Table S5. Experimental and predicted data for the response variables obtained from the central composite design for sea bass tailfin.

RSM				TAILFIN					
Run	pH	Tª (°C)	Time (min)	Protein (mg)		TEAC (μM Trolox Eq)		ORAC (μM Trolox Eq)	
				experimental	predicted	experimental	predicted	experimental	predicted
1	7	20	10	98	103	323	322	605	655
2	7	40	5	130	116	399	414	660	736
3	7	40	10	130	132	422	416	918	832
4	7	40	10	134	132	345	416	723	832
5	7	40	15	145	159	592	545	1179	1091
6	7	60	10	231	226	512	481	1177	1116
7	4	20	5	121	130	357	353	597	623
8	4	20	15	105	99	400	424	829	801
9	4	40	10	146	133	454	401	806	753
10	4	60	5	181	185	392	407	816	776
11	4	60	15	264	269	601	619	1253	1348
12	10	20	5	102	97	315	305	662	569
13	10	20	15	103	99	363	355	663	707
14	10	40	10	116	127	357	378	749	791
15	10	60	5	168	174	445	429	914	945
16	10	60	15	298	289	608	620	1501	1477

Regression equations provided by Response Surface Methodology and Statgraphics Centurion XVI.I for sea bass tailfins.

Equation S13: Protein = 289,54 - 7,1171*pH - 6,84346*Tª (ºC) - 14,9605*Time (min) - 0,195421*pH^2 + 0,0872708*pH*Tª (ºC) + 0,536917*pH*Time (min) + 0,080778*Tª (ºC)^2 + 0,284237*Tª (ºC)*Time (min) + 0,204248*Time (min)^2

Equation 14: TEAC = 405,904 + 28,7305*pH + 1,25441*Tª (ºC) - 49,57*Time (min) - 2,91966*pH^2 + 0,292521*pH*Tª (ºC) - 0,345917*pH*Time (min) - 0,0352047*Tª (ºC)^2 + 0,351362*Tª (ºC)*Time (min) + 2,55232*Time (min)^2

Equation 15: ORAC = 786,501 + 70,0734*pH - 15,4249*Tª (ºC) - 64,1887*Time (min) - 6,73596*pH^2 + 0,930187*pH*Tª (ºC) - 0,67025*pH*Time (min) + 0,132328*Tª (ºC)^2 + 0,985637*Tª (ºC)*Time (min) + 3,24746*Time (min)^2

Table S6. Reproducibility of the results according to the coefficient of variation from the values obtained in the central points of the central composite design model for sea bass side streams.

Sea bass side streams	Protein			TEAC			ORAC		
	average	SD	CV	average	SD	CV	average	SD	CV
Muscle	544,50	6,36	1,17	1288,90	94,86	7,36	2947,29	156,52	5,31
Head	174,89	19,95	11,41	636,01	20,33	3,20	1360,30	25,99	1,91
Viscera	116,10	1,78	1,53	490,93	23,24	4,73	1340,18	140,84	10,51
Skin	159,44	9,32	5,85	391,82	15,31	3,91	717,92	44,65	6,22
Tailfin	131,80	2,47	1,88	383,53	54,35	14,17	820,31	138,00	16,82

SD: Standard Desviation; CV: Coefient of Variation
